# Squalane as a Promising Agent Protecting UV-Induced Inhibition of Collagen Biosynthesis and Wound Healing in Human Dermal Fibroblast

**DOI:** 10.3390/molecules30091964

**Published:** 2025-04-29

**Authors:** Katarzyna Wolosik, Magda Chalecka, Gabriela Gasiewska, Jerzy Palka, Arkadiusz Surazynski

**Affiliations:** 1 Independent Cosmetology Laboratory, Medical University of Bialystok, Kilinskiego 1, 15-089 Bialystok, Poland; katarzyna.wolosik@umb.edu.pl; 2 Department of Medicinal Chemistry, Medical University of Bialystok, Kilinskiego 1, 15-089 Bialystok, Poland; magda.chalecka@umb.edu.pl (M.C.); gabriela.gasiewska@sd.umb.edu.pl (G.G.); jerzy.palka@umb.edu.pl (J.P.)

**Keywords:** UVA radiation, fibroblast, squalane, collagen biosynthesis, wound healing

## Abstract

Squalane, a highly stable derivative of squalene, has received attention for its potential application in dermatology and cosmetics due to its biocompatibility, moisturizing properties, and antioxidant activity. This study investigates the effects of squalane on UVA-induced oxidative stress, inflammation, deregulation of collagen metabolism, and some growth signaling pathways in human dermal fibroblasts (HDFs). It has been found that squalane at concentrations of 0.005–0.015% counteracted the UVA-induced inhibition of oxidative stress, collagen biosynthesis, prolidase activity, expression of the β1-integrin receptor, insulin-like growth factor-I receptor (IGFR), transforming growth factor-β (TGF-β), phosphorylated kinases ERK1/2, and increase in the expression of p38 kinase in HDFs. Moreover, squalane at the studied concentrations counteracted UVA-induced increase in the expression of NF-κB and COX-2 in HDFs, suggesting its anti-inflammatory activity. Interestingly, squalane augmented the UVA-induced expression of nuclear factor erythroid 2-related factor 2 (Nrf2). The functional significance of squalane activities was found in a model of wound healing in HDFs. Squalane at the studied concentrations stimulated fibroblast migration, facilitating the repair process following exposure of the cells to UVA radiation. These results demonstrate the ability of squalane to counteract UVA-induced cell damage and suggest its potential to support skin regeneration, highlighting its application in anti-aging, post-sun repair, and regenerative care formulations.

## 1. Introduction

### 1.1. The Impact of UV Radiation on Human Dermal Fibroblast’s Metabolism

Solar radiation is a key factor accelerating skin aging by disrupting cellular metabolism. UVA-irradiated human dermal fibroblasts (HDF) represent an experimental model to study metabolic consequences of solar radiation on skin cells. The mechanism of the harmful effect of UV radiation involves the induction of oxidative stress by the production of reactive oxygen species (ROS) and DNA damage. These processes contribute to the deregulation of cell metabolism, inhibition of collagen biosynthesis, and induction of apoptosis [1,2].

Collagen metabolism is impaired at both the transcriptional and post-transcriptional levels. At the transcriptional level, UVA induces the expression of NF-κB (nuclear factor kappa B), which acts as an inhibitor of collagen gene transcription [3]. Post-transcriptionally, it activates matrix metalloproteinases (MMPs) that degrade collagen extracellularly [4] and affects prolidase activity, an enzyme responsible for supplying proline from collagen degradation products (imidodi and imidotri peptides) for collagen resynthesis [5].

Collagen also plays a crucial role in regulating cellular metabolism as a ligand of integrin receptors. These receptors participate in signaling pathways that control collagen biosynthesis and prolidase activity [5]. Both processes are stimulated by insulin-like growth factor-I (IGF-I), one of the most potent stimulators of collagen biosynthesis, acting by a specific IGF-I receptor (IGFR) [6]. Under physiological conditions, the activation of β1-integrin and IGF-I receptors initiates a cascade of mitogen-activated protein kinase (MAPK) signaling pathways, including extracellular signal-regulated kinases (ERK-1 and ERK-2) [5]. Therefore, deregulation of collagen metabolism affects the key cell signaling pathways.

It is well established that ROS downregulate the expression of MAPK/ERK1/2 while promoting the activity of stress-activated kinases such as JNK (c-Jun N-terminal kinase) and p38. The effectors of MAP kinases include transcription factors such as c-Jun and c-Fos, which form the activating protein-1 (AP-1) complex. AP-1 plays a crucial role in collagen metabolism regulation by inhibiting pro-collagen type I gene expression and the transforming growth factor beta (TGF-β) signaling pathway [7,8].

Chronic UVA exposure induces inflammation in the skin, leading to the release of cytokines and proinflammatory factors, including NF-κB [4,9,10]. Activated NF-κB plays a key role in regulating the expression of various genes involved in immune and inflammatory responses, including cyclooxygenase-2 (COX-2), an enzyme associated with the inflammatory process [11]. These data show the complexity of metabolic processes that are affected by UVA radiation.

### 1.2. Squalane and Squalene: Structural and Functional Distinctions in Dermatological Applications

**Figure 1:**
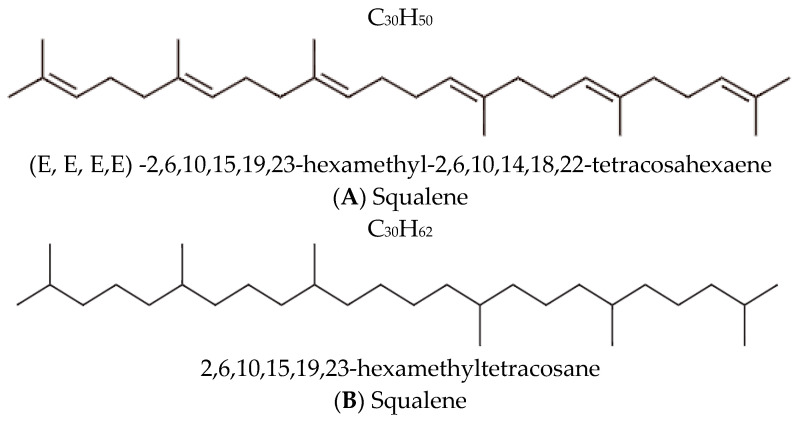
Chemical structures and nomenclature of (**A**) squalene and its saturated derivative (**B**) squalane [14,15].

Squalane and squalene are structurally related lipids, yet their distinct chemical properties significantly influence their utility in dermatological and cosmetic formulations. Squalene (C_30_H_50_) is an unsaturated hydrocarbon with six double bonds (Figure 1) and a major component of human sebum. It plays a crucial role in maintaining the skin’s hydrolipid barrier and protecting against environmental factors [12]. However, its high susceptibility to oxidation severely limits its stability, compromising both its efficacy and the shelf life of the formulations in which it is included [13]. Figure 1 illustrates the chemical structures of both squalene and squalane.

Squalane is an organic aliphatic hydrocarbon with a branched structure, chemically derived from squalene through the saturation of all its double bonds. This structural transformation yields a fully saturated molecule, C_30_H_62_, formally named 2,6,10,15,19,23-hexamethyltetracosane [15].

Squalane, a hydrogenated derivative of squalene, addresses the limitations of squalene by being fully saturated, rendering it highly resistant to oxidative degradation. This enhanced stability preserves its moisturizing and antioxidant properties over extended periods, making it an ideal choice for dermatological applications [13].

In addition to its chemical stability, squalane exhibits superior biocompatibility due to its saturated, aliphatic structure. It penetrates the epidermis efficiently without clogging pores, qualifying it as a non-comedogenic. These properties make squalane suitable for a wide spectrum of skin types, including sensitive and acne-prone skin [13].

The marked differences between squalene and squalane in terms of stability, biocompatibility, and overall efficacy underscore their respective roles in dermatology. Squalane’s stability and compatibility with skin enable its use in applications aimed at enhancing hydration, supporting collagen synthesis, and mitigating oxidative stress, which are essential for maintaining healthy skin and addressing signs of aging [15].

Given the susceptibility of dermal fibroblasts to UVA-induced damage and the critical role of collagen metabolism in maintaining skin integrity, this study aims to investigate whether Sq can counteract the UVA-induced inhibition of collagen biosynthesis and wound healing in HDFs. The study presents an analysis of the effects of Sq on cell viability, collagen biosynthesis, prolidase activity, and key signalling pathways related to collagen metabolism and inflammation. In addition, the functional effects of Sq are evaluated in a scratch wound healing assay. By investigating both molecular mechanisms and cellular responses, our findings highlight the potential of Sq as a bioactive compound in skin care formulations targeting UV protection, regeneration, and anti-aging.

## 2. Results and Discussion

### 2.1. Effect of Squalane on Viability of UVA-Irradiated Human Dermal Fibroblasts

The effects of different concentrations of squalane (Sq) on cell viability were studied in HDFs. At concentrations of 0.005%, 0.01%, and 0.015%, Sq did not significantly affect cell viability, compared to the control cells, untreated cells maintained in phosphate-buffered saline (PBS) and not exposed to UVA radiation or Sq treatment. However, at concentrations of 0.02% and 0.025%, Sq reduced the cell viability to 78% and 74% of the values found in the control cells, respectively. Although representative microscopic images of HDFs were not shown, direct real-time observations under the microscope revealed that, at the highest tested concentration of Sq (0.025%), some fibroblasts appeared less adherent to the culture surface and exhibited slightly reduced confluence compared to the cells cultured in lower concentrations of Sq. These subtle morphological features may indicate early signs of altered cell behavior, such as reduced proliferation or adhesion capacity.

**Figure 2:**
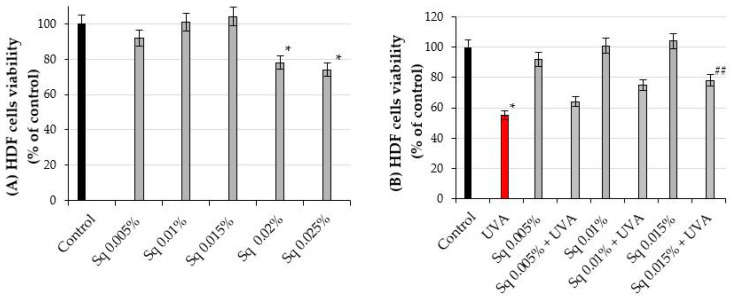
(**A**) The viability of HDF treated with squalane (Sq) at concentrations of 0.005%, 0.01%, 0.015%, 0.02%, and 0.025%. (**B**) The viability of HDF irradiated with UVA and treated with Sq at concentrations of 0.005%, 0.01%, and 0.015% compared to the control (PBS-treated cells without exposure to UVA or squalane) and to the UVA-only group (cells irradiated with UVA without squalane). The mean ± standard error (SEM) values from the experiments were performed in triplicates. * Statistically significant differences at *p* < 0.05 compared to the control. ## Statistically significant difference at *p* < 0.05 compared to the UVA-only group (red bar).

Consequently, these concentrations (0.02% and 0.025%) were excluded from further experiments (Figure 2A, Table S1).

In our previous studies, we found that the exposure of fibroblasts to UVA radiation affects the cell viability in a dose-dependent manner [16]. Based on these findings, a UVA dose of 10 J/cm^2^ was selected for the experiments, as it corresponded approximately to the IC_50_ value for cell viability in the experimental model. The exposure of HDFs to UVA radiation at a dose of 10 J/cm^2^ resulted in a significant decrease in cell viability to 55% of the control value (*p* < 0.05). However, pre-treatment of the cells with Sq at concentrations of 0.005%, 0.01%, and 0.015% evoked a protective effect against UVA-induced HDF cytotoxicity. As shown in Figure 2B, the viability of UVA-irradiated HDF pre-treated with Sq at concentrations of 0.005%, 0.01%, and 0.015% reached 64%, 75%, and 78% of the values found in the control cells, respectively. Compared to the viability of UVA-treated cells (55% of control), only the 0.015% Sq concentration resulted in a statistically significant improvement in cell viability (*p* < 0.05). Figure 2B and Table S1 show that Sq exerted a protective effect against UVA-induced cytotoxicity, with the most pronounced effect observed at 0.015%. However, a clear concentration-dependent pattern was not statistically confirmed.

**Figure 3:**
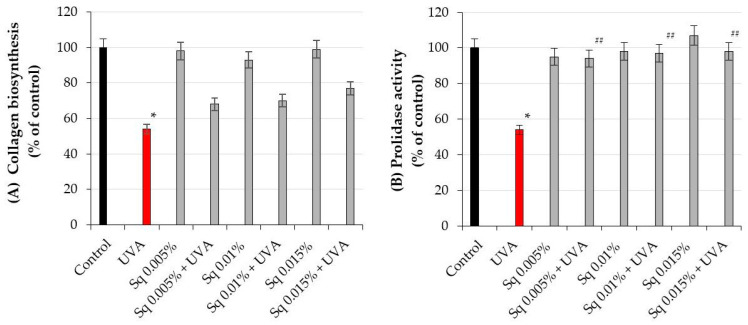
(**A**) Collagen biosynthesis and (**B**) prolidase activity in HDF irradiated with UVA and treated with squalane (Sq) at concentrations of 0.005%, 0.01%, and 0.015%. The results are compared to the control (PBS-treated cells without exposure to UVA or squalane) and to the UVA-only group (cells irradiated without squalane). The mean ± standard error (SEM) values from the experiments were performed in triplicate. * Statistically significant differences at *p* < 0.05 compared to the control. ## Statistically significant difference at *p* < 0.05 compared to the UVA-only group (red bar).

As shown in Figure 3B, the viability of HDFs in this experiment was found at 64%, 75%, and 78% of the values found in the cells exposed only to UVA radiation, respectively. These differences were statistically significant (*p* < 0.05). Figure 3B and Table S1 show that Sq mitigates the UVA-induced reduction in HDF viability in a concentration-dependent manner, with the most protective effect at a concentration of 0.015%.

While Sq demonstrates cytoprotective properties at concentrations up to 0.015%, higher concentrations may have adverse effects, underscoring the importance of dose optimization in its potential application as a UV-protective agent. Prolonged exposure of cells to UV radiation induces the generation of ROS, leading to oxidative stress. This process affects the redox balance; integrity of biological membranes; impairs collagen biosynthesis; and induces the damage of cellular components, including DNA, proteins, and lipids, in skin cells such as fibroblasts and keratinocytes [17].

It was considered that trace impurities might contribute to UV protection, but the current literature does not report any significant UV-absorbing activity from such impurities in highly purified squalane preparations. Therefore, based on both the product specification and existing scientific evidence, we conclude that UV protection observed in our study is due to the biological activity of Sq.

### 2.2. The Effect of Sq on UVA-Induced Inhibition of Collagen Biosynthesis and Prolidase Activity in HDsF

It is well established that UV-induced skin damage is due to ROS-induced oxidative stress, which disrupts collagen metabolism [18]. The experiment conducted in this study shows that UVA radiation at the studied dose contributed to the decrease in collagen biosynthesis and prolidase activity, an important enzyme in recycling proline for collagen resynthesis. Prolidase catalyzes the final step of collagen degradation and plays a key role in providing proline for collagen synthesis and cell growth [5]. The inhibition of collagen biosynthesis by UVA radiation was counteracted by Sq at studied doses (Figure 3A). When the cells were exposed to UVA radiation, collagen biosynthesis was reduced to 54% of the control value. However, Sq at concentrations of 0.05%, 0.01%, and 0.015% counteracted the destructive effect of UV on this process to 68%, 70%, and 77% of the control values, respectively (Figure 3A, Table S2). Although these results suggest a possible upward trend, the differences between the Sq-treated and UVA-treated cells were not statistically significant (*p* > 0.05). Therefore, the observed increase in collagen biosynthesis should be interpreted as a non-significant trend rather than a confirmed effect. Similarly, Sq counteracted the UVA-induced inhibition of prolidase activity (Figure 3B). In UVA-irradiated HDFs, the prolidase activity was reduced to 54% of the control, while Sq restored the activity to 94%, 97%, and 98% of the control, respectively (Figure 3B, Table S3). It suggests a protective effect of Sq on UVA-induced deregulation of collagen metabolism in HDFs. In fact, statistical analysis, with a Spearman correlation coefficient of 0.88 (*p* < 0.01), showed a positive correlation between prolidase activity and collagen biosynthesis in UVA- and Sq-treated cells. This highlights the potential of Sq as a bioactive compound to restore dermal homeostasis by enhancing the enzymatic activity of prolidase. providing a substrate (proline) for collagen production. These findings provide new insights into the biochemical pathways modulated by Sq and its utility in mitigating UVA-induced dermal damage.

These results are consistent with our previous study showing the protective effect of *Amaranthus cruentus* seed oil on the deregulation of collagen biosynthesis and prolidase activity in UVA-irradiated fibroblasts [19].

While Sq is slightly less effective than *Amaranthus cruentus* seed oil in restoring the UVA-induced inhibition of collagen biosynthesis [19], its ability to maintain prolidase activity in these conditions at a near-control level highlights its significant protective role. These discoveries open up new possibilities for incorporating Sq into dermatological formulations not only to reduce UVA-induced skin damage but also as a regenerative or anti-aging agent. Our previous studies show that other bioactive preparations, such as *Amaranthus cruentus* seed oil, could provide similar protection [20].

### 2.3. The Effect of Sq on the UVA-Induced Inhibition of Cell Signaling in HDFs

**Figure 4:**
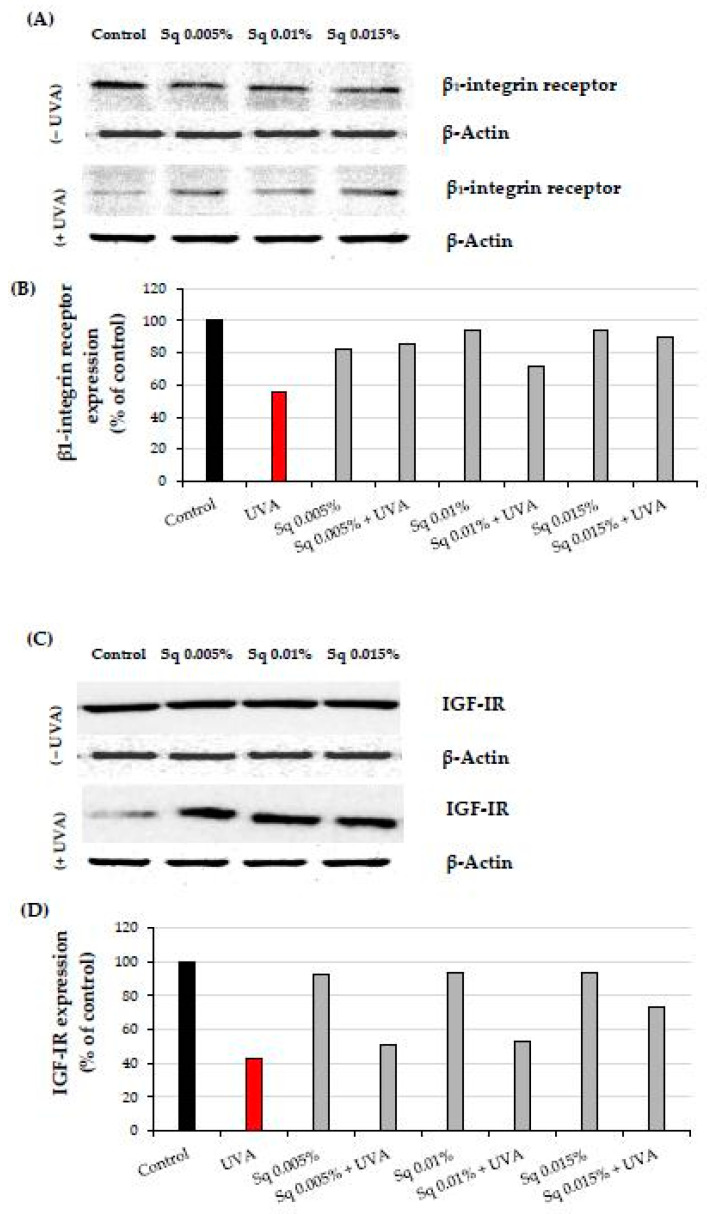
(**A**) Western blot and (**B**) densitometry (completed using ImageJ^®^ 1.8.0) for β1-integrin receptor expression with actin as a loading control, and (**C**) Western blot and (**D**) densitometry for IGF-IR expression with actin as a loading control in HDF irradiated with UVA and treated with squalane (Sq) at concentrations of 0.005%, 0.01%, and 0.015%. The results are compared to the control (PBS-treated cells without exposure to UVA or squalane). Data were obtained from 3 pooled homogenates.

The β1-integrin receptor plays a crucial role in cellular signaling pathways that upregulate collagen biosynthesis and prolidase activity [4]. UVA radiation significantly reduced β1-integrin expression in HDFs, impairing these processes. However, Sq treatment effectively reduced this UVA-dependent deleterious effect, suggesting its protective role in this process (Figure 4A and Figure S1). This is consistent with previous findings that β1-integrin activation is essential for initiating collagen biosynthesis and maintaining prolidase activity [5].

The insulin-like growth factor I receptor (IGF-IR) is another key stimulator of collagen biosynthesis [6]. UVA-induced downregulation of IGF-IR disrupted this process. Sq at 0.01% and 0.015% concentrations prevented IGF-IR downregulation in HDFs, particularly when used with higher concentrations (Figure 4B and Figure S1). These findings suggest the mechanism (IGF-IR signaling) by which Sq mitigates UVA-induced impairment of collagen metabolism.

**Figure 5:**
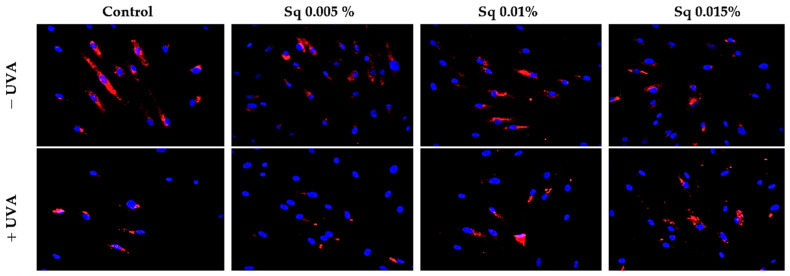
Expression of ERK 1/2 kinase (ERK 1/2) visualized by immunofluorescence staining in HDF irradiated with UVA and treated with squalane (Sq) at concentrations of 0.005%, 0.01%, and 0.015%. The results are compared to the control (PBS-treated cells without exposure to UVA or squalane). Blue staining indicates cell nuclei; red staining indicates p-ERK 1/2 expression. Images were obtained at 20× magnification. Note: The antibody used detects the total ERK1/2, not its phosphorylated form.

Both β1-integrin and IGF-IR are known to activate mitogen-activated protein kinases (MAPKs), including ERK1/2, which are involved in cellular repair and survival mechanisms. UVA radiation is known to disrupt MAPK signaling, including the ERK1/2 pathway. In our study, immunofluorescence staining suggested a decrease in ERK1/2 signaling following UVA exposure, whereas Sq treatment appeared to increase this signaling (Figure 5). However, as the antibody used detects total ERK1/2 and not its phosphorylated form, no conclusions can be drawn regarding phosphorylation.

The study confirms a positive correlation between receptor expression (β_1_-integrin and IGF-IR), prolidase activity, and collagen biosynthesis. It suggests that the molecular mechanisms of the protective effect of Sq on UVA-induced deregulation of collagen metabolism may involve the modulation of MAPK signaling, including ERK1/2, although further studies using phosphorylation-specific assays would be required to confirm the activation status of these kinses. Although our earlier studies demonstrated a similar mechanism for the protective effects of *Amaranthus cruentus* seed oil against UVA-induced cell damage, the stability of Sq makes it a unique candidate for protecting collagen metabolism from UVA-induced oxidative stress.

**Figure 6:**
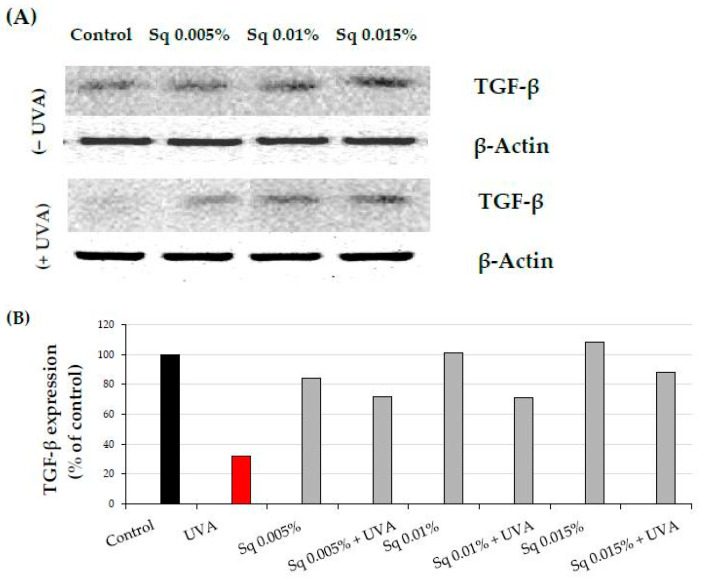

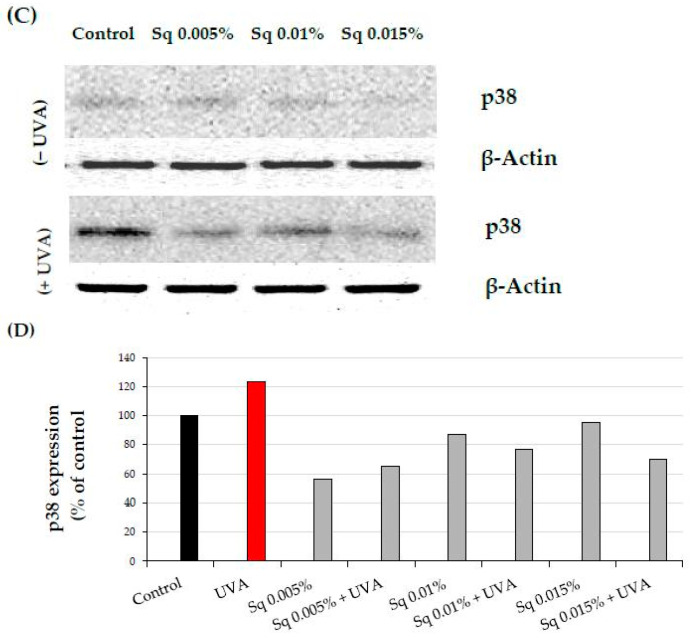
(**A**) Western blot and (**B**) densitometry (completed using ImageJ^®^ 1.8.0) for TGF-β1 expression with actin as a loading control, and (**C**) Western blot and (**D**) densitometry for p38 protein expression with actin as a loading control in HDF irradiated with UVA and treated with squalane (Sq) at concentrations of 0.005%, 0.01%, and 0.015%. The results are compared to the control (PBS-treated cells without exposure to UVA or squalane). Data were obtained from 3 pooled homogenates.

Activation of the β1-integrin receptor and IGF-IR is known to trigger not only MAPKs signaling but also the transforming growth factor β (TGF-β) pathway [8,9]. MAPK signaling is critical for cellular functions, and disruption of this pathway is linked to oxidative stress. UVA-induced ROS reduces the expression of phosphorylated ERK1/2 (p-ERK1/2) but enhances the expression of p38, a stress-activated kinase. In the current study, we observed that UVA exposure significantly reduced the expression of TGF-β_1_ (Figure 6A,B and Figure S2A), while it increased the expression of p38 (Figure 6C,D and Figure S2B). Sq at the studied concentrations evokes a protective effect on UVA-induced decrease in TGF-β_1_ and increase in p-38 expression. This aligns with existing evidence that antioxidants derived from natural sources, such as green tea, pomegranate extract, or *Amaranthus cruentus* seed oil, can counteract oxidative stress and restore cellular signaling pathways affected by UVA [19,21,22].

### 2.4. The Effect of Sq on UVA-Induced Inflammation in HDF

**Figure 7:**
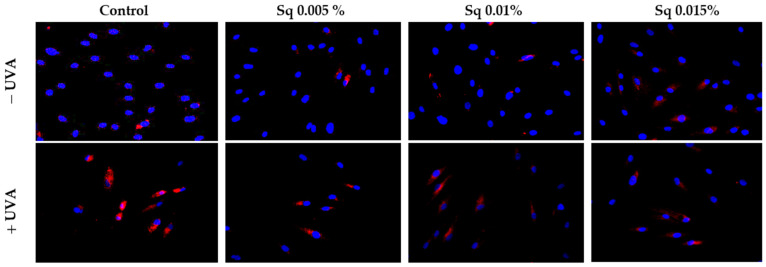
Expression of NF-κB (visualized by immunofluorescence staining) in HDF irradiated with UVA and treated with squalane (Sq) at concentrations of 0.005%, 0.01%, and 0.015%. The results are compared to the control (PBS-treated cells without exposure to UVA or squalane). Blue staining indicates nuclei, and red staining indicates NF-κB expression. The images were obtained at 20× magnification. Note: The antibody used detects the total NF-κB, not its phosphorylated form.

Chronic UV exposure leads to skin damage through inflammatory processes involving cytokines and transcription factors, including nuclear factor kappa B (NF-κB), a known inhibitor of gene expression for collagen type I subunits [20]. Immunofluorescence staining suggested that UVA exposure increased the NF-κB signal in HDF and that treatment with Sq at 0.01% and 0.015% appeared to reduce this signal. However, as no separate quantification or validation of nuclear staining was performed, no definitive conclusions regarding NF-κB translocation can be drawn (Figure 7).

**Figure 8:**
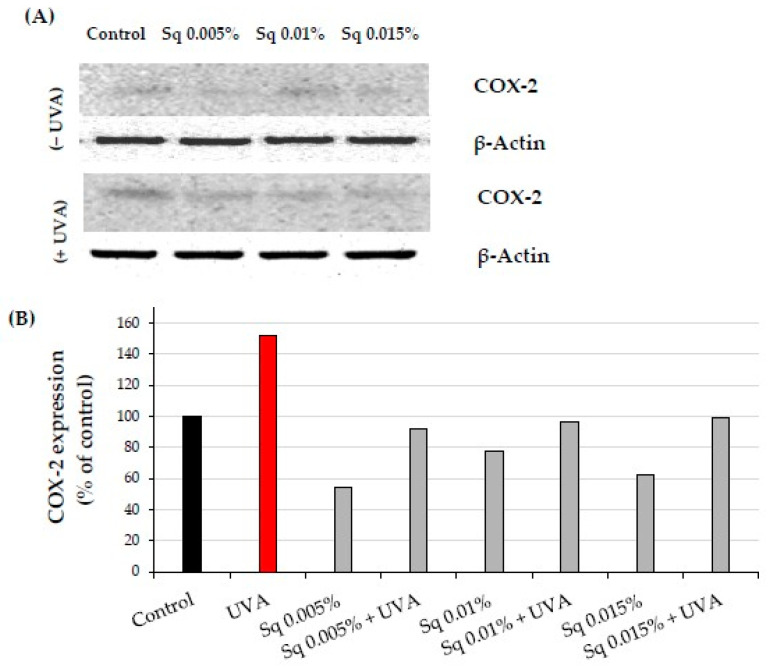
(**A**) Western blot and (**B**) densitometry (completed using ImageJ^®^ 1.8.0) for COX-2 expression with actin as a loading control in HDF irradiated with UVA and treated with squalane (Sq) at concentrations of 0.005%, 0.01%, and 0.015%. The results are compared to the control (PBS-treated cells without exposure to UVA or squalane). Data were obtained from 3 pooled homogenates.

NF-κB plays a key role in regulating the expression of genes involved in immune and inflammatory responses, including cyclooxygenase-2 (COX-2), critical enzyme in the inflammatory cascade. We found that UVA exposure at a dose of 10 J/cm^2^ caused an increase in COX-2 expression in fibroblasts. This phenomenon is consistent with a similar finding by Vostálová et al. in a mouse skin model exposed to 20 J/cm^2^ of UVA [11]. Treatment of HDF with Sq reversed this effect close to the values of the control cells, particularly at Sq concentrations of 0.01% and 0.015% (Figure 8A,B and Figure S3). These results suggest that Sq may reduce NF-κB-associated inflammatory signaling, as indicated by reduced NF-κB and COX-2 protein expression. However, the suppression of NF-κB activation should be confirmed using additional techniques such as nuclear–cytoplasmic fractionation or phosphorylation-specific assays.

The anti-inflammatory effects of Sq are likely attributed to its ability to stabilize the cellular redox balance, reducing ROS and preventing the activation of NF-κB. In its inactive state, NF-κB is regulated in the cytoplasm by the inhibitor protein IκB. Upon UV-induced oxidative stress, IκB is degraded, allowing NF-κB to translocate to the nucleus and initiate the transcription of pro-inflammatory genes, including COX-2 [23]. Sq may potentially modulate this pathway, although our study did not directly assess the IκB status or nuclear localization of NF-κB. The observed changes in fluorescence are suggestive of altered NF-κB expression but not conclusive translocation to the nucleus.

A key cytoprotective response to oxidative damage is the activation of nuclear factor erythroid 2–related factor 2 (Nrf2), a transcription factor that regulates genes encoding antioxidant enzymes and proteins. Nrf2 plays a vital role in protecting the skin from oxidative damage caused by environmental factors, including UV radiation. While UVB inhibits Nrf2 signaling in keratinocytes and melanocytes, UVA stimulates its activation in fibroblasts, which are more susceptible to oxidative damage due to deeper UVA penetration [24]. Nrf2 activation involves its translocation to the nucleus, where it upregulates antioxidant and detoxifying enzymes. Natural compounds like vitamin C, rutin, quercetin, sea buckthorn oil, and *Amaranthus cruentus* seed oil have been shown to enhance Nrf2 activation [19,25,26]. Gęgotek et al. [25] demonstrated that sea buckthorn oil increased Nrf2 expression in UVA-irradiated keratinocytes and fibroblasts, enhancing their antioxidant capacity and preventing redox disturbances.

**Figure 9:**
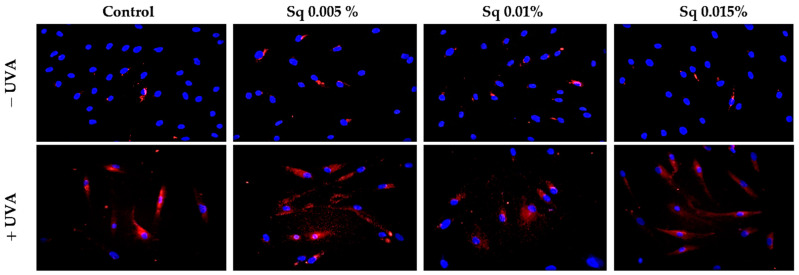
Expression of Nrf2 (visualized using immunofluorescence staining) in HDF irradiated with UVA and treated with squalane (Sq) at concentrations of 0.005%, 0.01%, and 0.015%. The results are compared to the control (PBS-treated cells without exposure to UVA or squalane). Blue staining indicates the nuclei, and red staining represents Nrf2 expression. Images were obtained at 20× magnification. Note: The antibody used detects the total Nrf2, not its phosphorylated form.

We observed that UVA exposure increased Nrf2 expression in HDF. The treatment of HDF with Sq enhanced Nrf2 expression and its nuclear translocation (Figure 9). These findings suggest that Sq, similar to sea buckthorn oil and *Amaranthus cruentus* seed oil, intensify UVA-induced Nrf2 activation, increasing the cellular antioxidant response and protecting against UVA-induced oxidative stress. Therefore, Sq could be considered as an effective agent for boosting the skin’s natural defenses against photooxidative damage.

### 2.5. The Effect of Sq on Wound Healing in UVA-Irradiated HDFs

**Figure 10:**
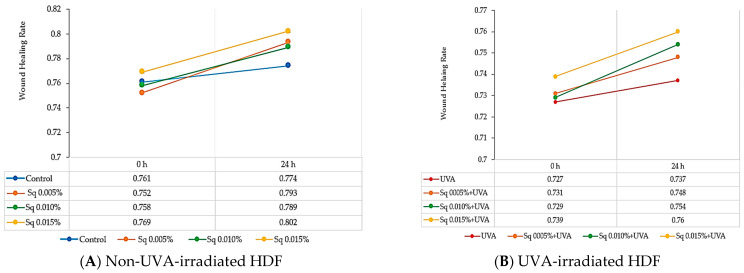
Wound healing rate in HDF treated with squalane (Sq) at concentrations of 0.005%, 0.01%, and 0.015%. (**A**) Non-UVA-irradiated HDF after 24 h of treatment. (**B**) UVA-irradiated HDFs after 24 h of treatment. The results are compared to the control (PBS-treated cells without exposure to UVA or squalane). The wound closure rate was quantified and analyzed using ImageJ^®^ 1.8.0 software with the Wound Healing Size Tool extension.

The skin serves as a protective barrier, safeguarding the body from external factors, and any damage to its integrity initiates repair processes. Fibroblasts play a pivotal role in wound healing, spanning from the initial inflammatory phase to the final production of extracellular matrix components. The effects of Sq on fibroblast migration and proliferation were assessed using the scratch test method, with the data analyzed via fluorescence microscopy and Image ImageJ^®^ 1.8.0 software equipped with the Wound Healing Size Tool extension. The wound healing rate was calculated and represented in graphical form. The results indicate that Sq significantly promoted fibroblast proliferation and migration in UVA-irradiated HDF. Sq’s effect on wound closure was positive, enhancing the wound healing rate over time (Figure 10B). Although Sq demonstrated a moderate protective effect on wound healing in cells exposed to UVA radiation (Figure 10A), the data suggest the potential role of Sq in mitigating the adverse effects of UVA on fibroblast migration and wound closure.

## 3. Materials and Methods

### 3.1. Materials

Neossance™ Squalane, manufactured by Aprinnova, LLC (Emeryville, CA, USA), was used in the studies described in this work. According to the manufacturer, the product is derived from renewable sugarcane certified by Bonsucro.

Product Specifications:
Product Name (IUPAC): Neossance Squalane (2,6,10,15,19,23-hexamethyltetracosane)
Purity (%): Min. 92
Density (g/cm^3^): Min. 0.806–Max. 0.811
Iodine Number (g I_2_/100 g): Max. 2
Acid Value (mg KOH/g oil): Max. 0.5
Odor: Nearly odorless
Color: Colorless

According to the supplier’s specification (Aprinnova), the Neossance Squalane used in our study has a minimum GC purity of 92% for squalene and 99% when including its derivative, with no significant unsaturated or aromatic compounds indicated. Given that squalene is a fully saturated hydrocarbon (C_30_H_62_), its UV absorbance in the UVA and UVB range is well documented to be negligible due to the absence of conjugated double bonds or chromophores [27,28].

### 3.2. Methods

#### 3.2.1. Cell Culture

Human dermal fibroblasts (HDFs) were obtained from the American Type Culture Collection (Manassas, VA, USA) and cultured in Dulbecco’s Modified Eagle’s Medium (DMEM) (PAN™ BIOTECH, Aidenbach, Germany) supplemented with 10% fetal bovine serum (FBS) (Gibco, Waltham, MA, USA), 50 U/mL of penicillin (Pen) (Gibco, Waltham, MA, USA), and 50 μg/mL of streptomycin (Strep) (Gibco, Waltham, MA, USA). The cells were maintained at 37 °C in a 5% CO_2_ atmosphere and grown on 100 mm dishes in 10 mL of complete medium, which was replaced 2–3 times per week. Cells at passages 8–10 were used for the experiments.

#### 3.2.2. Assessment of Cytotoxicity

The cytotoxic effect of UVA radiation and Sq on HDF were assessed by the methylthiazolyl tetrazolium (MTT) assay based on the method described by Carmichael et al. [29]. The assay is based on reducing the yellow tetrazolium salt (MTT) to purple formazan crystals by mitochondrial dehydrogenases active in viable cells. For the experiments, the fibroblasts were seeded in 6-well plates at an initial density of 3 × 10^5^ cells per well. When the cells reached approximately 80% confluence, the culture medium was removed, and all experiments were performed in triplicate. The concentrations of Sq studied were 0.005%, 0.01%, and 0.015%. After 30 min of incubation with squalane, the medium was aspirated, and the cells were rinsed with PBS. The cells were then exposed to UVA light using a Bio-Link Crosslinker BLX 365 (Vilber Lourmat, Eberhardzell, Germany) at a dose of 10 J/cm^2^ (365 nm) in 1 mL cold PBS (4 °C). After irradiation, the PBS was replaced with fresh DMEM. The cells were incubated at 37 °C for 24 h. At the end of the incubation period, the culture medium was removed, and the cells were washed twice with pre-warmed PBS. The cells were then incubated with MTT (0.5 mg/mL, 1.0 mL per well) dissolved in PBS at 37 °C for 1 h. After incubation, the MTT solution was aspirated and formazan crystals dissolved in DMSO (1.0 mL per well). The absorbance of the resulting solution was measured at 570 nm using a spectrophotometer. Cell viability was expressed as a percentage of the values found in the control cells. The control cells were incubated in the same conditions in PBS without exposure to UVA radiation or treatment with Sq. The cell viability, collagen biosynthesis, enzyme activity, and expression of proteins were compared to the untreated control cells.

#### 3.2.3. Collagen Biosynthesis Assay

HDFs were cultured in 100 mm plates at a density of 1 × 10^6^ cells per plate. At confluence, the cells were treated as described in Section 3.2.2. Collagen biosynthesis was assessed by the incorporation of radioactive 5-[^3^H]-proline (5 μCi/mL; Hartmann Analytic, Germany) into bacterial collagenase-sensitive proteins. The procedure was performed according to the method described by Peterkofsky [30]. After 24 h of incubation, the cells were rinsed with PBS (pH 7.4), resuspended in PBS containing 10 mM proline, and stored at −80 °C until further analysis. Purified *Clostridium histolyticum* collagenase (Sigma-Aldrich, St. Louis, MO, USA) was used for collagen digestion. Radiometric measurements were performed with a Tri-Carb 2810 TR liquid scintillation analyzer (PerkinElmer, Waltham, MA, USA). Data were normalized to total protein synthesis and expressed as a percentage of the control, providing quantitative information on collagen biosynthesis in the context of the experimental conditions.

#### 3.2.4. Prolidase Activity Assay

HDF cells were cultured on 100 mm plates at a density of 1 × 10^6^ cells per plate. At confluence, the cells were treated as described in 3.2.2. Cells were harvested, and prolidase activity was measured using the Myara [31] protocol after 24 h of incubation. The total protein concentration in the samples was determined by the Lowry method [32]. Prolidase activity was expressed as nanomoles of proline released per minute from the synthetic substrate (Gly-Pro). This was normalized to the protein content (milligrams) in the cell homogenate supernatant.

#### 3.2.5. Western Blot Analysis

HDFs were cultured on 100 mm plates at a density of 2 × 10^6^ cells per plate. When the cells reached confluence, they were treated with Sq and UVA, as described in Section 3.2.2. After 24 h of incubation, the cells were lysed using cell lysis buffer supplemented with a protease/phosphatase inhibitor cocktail. Protein concentrations were determined by the Lowry method [32]. Proteins were separated by SDS-PAGE using the Laemmli method [33]. After electrophoresis, the gels were rinsed with cold Towbin buffer (25 mM Tris, 192 mM glycine, 20% methanol (*v*/*v*), and 0.025–0.1% SDS, pH 8.3), and the proteins were transferred to 0.2 µm nitrocellulose membranes using a Trans-Blot system (Bio-Rad, Hercules, CA, USA). The transfer was performed at 200 mA for 3 h in a freshly prepared Towbin buffer at a temperature of 4–8 °C. The membranes were blocked with 5% non-fat dry milk (NFDM) for 1 h at room temperature. After blocking, the membranes were washed three times with 20 mL TBS-T buffer (20 mM Tris, 150 mM NaCl, and 0.1% Tween^®^ 20). The membranes were then incubated overnight at 4 °C with primary antibodies at a dilution of 1:1000. After incubation, the membranes were washed three times with 20 mL TBS-T and treated with horseradish peroxidase (HRP)-conjugated secondary antibodies at a 1:3000 dilution in 5% NFDM for 1 h at room temperature. After incubation, the membranes were washed three more times with 20 mL TBS-T, and protein bands were visualized using the BioSpectrum Imaging System UVP (Ultra-Violet Products Ltd., Cambridge, UK).

#### 3.2.6. Protein Expression Visualization by Confocal Microscopy

HDFs were cultured in a black 96-well plate. At confluence, the cells were treated as in Section 3.2.2. After 24 h, the culture medium was removed, and the cells were fixed with a 3.7% formaldehyde solution for 10 min at room temperature. After fixing, the wells were washed once using 100 µL PBS per well. Permeabilization was performed with 0.1% Triton X-100 solution for 10 min. The plate was then washed twice with PBS, and 3% FBS was applied as a blocking agent for 30 min at room temperature. After blocking, the FBS solution was removed, and 50 µL of primary antibody (diluted 1:50 in 3% FBS) was added to each well. The plate was incubated for 1 h at room temperature. The wells were washed three times with PBS after incubation. Then, 50 µL of secondary antibody (1:1000 dilution) was added to each well, and the plate was incubated for 1 h in the dark to protect the fluorescence signal. After the removal of the secondary antibody, the wells were washed three times with PBS. For nuclear staining, 100 µL of PBS containing 2 µg/mL Hoechst 33342 was added to each well. Fluorescence was visualized using a BD Pathway 855 confocal laser scanning microscope (Bioimager, Becton Dickinson, Franklin Lakes, NJ, USA) equipped with AttoVision™ 1.6 software.

#### 3.2.7. Antibodies

For Western blot and immunofluorescence staining, the following primary antibodies were used: IGF-1 Receptor Rabbit mAb (1:1000), β_1_-Integrin Receptor Rabbit mAb (1:2000), and TGF-β Receptor 1 Rabbit Antibody (1:1000) purchased from Cell Signaling Technology (Danvers, MA, USA); MAPK (ERK1/2) Rabbit mAb (1:1000) and NF-κβ p65 Mouse Antibody (1:1000) were purchased from Becton Dickinson (Franklin Lakes, NJ, USA). COX-2 Mouse Antibody, p38 MAPK, and β-Actin Mouse Antibody were purchased from Santa Cruz Biotechnology Inc. (Dallas, TX, USA). Secondary HRP-conjugated antimouse or antirabbit antibodies diluted 1:5000 were from Sigma-Aldrich (St. Louis, MO, USA).

#### 3.2.8. Fibroblast Migration Using Scratch Assay

Confluent HDFs cultured in 6-well plates were subjected to a scratch assay by creating a wound using a sterile 200 µL pipette tip. Then, they were treated as described in Section 3.2.2. An inverted light microscope (Nikon, Minato, Tokyo, Japan) was used to observe and monitor the scratched area at 20× magnification. The migration of the fibroblasts was quantified using ImageJ^®^ 1.8.0 software with the Wound Healing Size Tool extension, and the rate of migration was calculated according to the following formula.

$$wound\;healing\;rate\;=\;\frac{original\;wound\;area\;-\;unhealed\;wound\;area}{original\;wound\;area}$$

## 4. Conclusions

This study demonstrates that Sq, at concentrations ranging from 0.005% to 0.015%, exerts a protective effect on disturbed metabolism in UVA-irradiated HDFs. Sq counteracted the UVA-induced decrease in cell viability, collagen metabolism, inflammation, and wound healing. These data suggest the potential use of Sq in skin care formulations aimed at UV protection, tissue regeneration, and anti-aging.

## Data Availability

The data presented in this study are available in the main text of this article or on request from the corresponding author.

## Supplementary Materials

The following supporting information can be downloaded at https://www.mdpi.com/article/10.3390/molecules30091964/s1: Table S1. The MTT test for HDF viability (A) treated with Sq at concentrations of 0.005%, 0.01%, 0.015%, 0.02%, and 0.025% vs. the control (B) irradiated with UVA and treated with Sq at concentrations of 0.005%, 0.01%, and 0.015% vs. the control. The mean values. standard deviation (SD), standard error (SEM), and median (Me) from the 3 experiments. * Statistically significant differences at *p* < 0.05 compared to the control. ^##^ Statistically significant differences at *p* < 0.05 compared to the UVA group. Table S2. Collagen biosynthesis measurements in HDF irradiated with UVA and treated with Sq at concentrations of 0.005%, 0.01%, and 0.015%. (1st: first measurement in sample. 2nd: second measurement in sample. Δ—difference between the 1st and 2nd measurements in the sample. * Statistically significant differences at *p* < 0.05 compared to the control. Table S3. Prolidase activity in HDF irradiated with UVA and treated with Sq at concentrations of 0.005%, 0.01%, and 0.015%. The mean values from the experiments performed in triplicate. * Statistically significant differences at *p* < 0.05 compared to the control. ^##^ Statistically significant difference at *p* < 0.05 compared to UVA. Figure S1. Western blot densitometry (plot measurement) results by gel analysis tool for Fiji ImageJ^®^ 2.16.0 for (A) β1-integrin (B) and IGF-IR receptors expression in UVA-irradiated HDFs in the presence of Sq at concentrations of 0.005%, 0.01%, and 0.015%. Figure S2 Western blot densitometry (plot measurement) results by the gel analysis tool for ImageJ^®^ 1.8.0 for (A) TGF-β1 and (B) p38 protein expression in UVA-irradiated HDF in the presence of Sq at concentrations of 0.005%, 0.01%, and 0.015%. Figure S3 Western blot densitometry (plot measurement) results by the gel analysis tool for ImageJ^®^ 1.8.0 for COX-2 protein expression in UVA-irradiated HDF in the presence of Sq at concentrations of 0.005%, 0.01%, and 0.015%.

## Abbreviations

The following abbreviations are used in this manuscript:

COX-2	Cyclooxygenase-2
ERK1/2	Extracellular Signal-Regulated Kinases 1/2
HDF	Human Dermal Fibroblasts
Sq	Squalane
UVA	Ultraviolet A Radiation
ROS	Reactive Oxygen Species
NF-κB	Nuclear Factor Kappa B
IGF-IR	Insulin-Like Growth Factor I Receptor
β1-integrin	Beta 1 Integrin
Nrf2	Nuclear Factor Erythroid 2–Related Factor 2
TEWL	Transepidermal Water Loss
MAPKs	Mitogen-Activated Protein Kinases
TGF-β	Transforming Growth Factor Beta

## References

[1] Yaar M., Gilchrest B.A. (2007). Photoageing: Mechanism, Prevention and Therapy. Br. J. Dermatol..

[2] Lavker R.M., Gerberick G.F., Veres D., Irwin C.J., Kaidbey K.H. (1995). Cumulative Effects from Repeated Exposures to Suberythemal Doses of UVB and UVA in Human Skin. J. Am. Acad. Dermatol..

[3] Chen L., Hu J.Y., Wang S.Q. (2012). The Role of Antioxidants in Photoprotection: A Critical Review. J. Am. Acad. Dermatol..

[4] Chen Q., Zhang H., Yang Y., Zhang S., Wang J., Zhang D., Yu H. (2022). Metformin Attenuates UVA-Induced Skin Photoaging by Suppressing Mitophagy and the PI3K/AKT/mTOR Pathway. Int. J. Mol. Sci..

[5] Surazynski A., Miltyk W., Palka J., Phang J.M. (2008). Prolidase-Dependent Regulation of Collagen Biosynthesis. Amino Acids.

[6] Baszanowska W., Misiura M., Oscilowska I., Palka J., Miltyk W. (2021). Extracellular Prolidase (PEPD) Induces Anabolic Processes through EGFR, Β1-Integrin, and IGF-1R Signaling Pathways in an Experimental Model of Wounded Fibroblasts. Int. J. Mol. Sci..

[7] Xia Z., Dickens M., Raingeaud J., Davis R.J., Greenberg M.E. (1995). Opposing Effects of ERK and JNK-P38 MAP Kinases on Apoptosis. Science.

[8] Verheij M., Bose R., Lin X.H., Yao B., Jarvis W.D., Grant S., Birrer M.J., Szabo E., Zon L.I., Kyriakis J.M. (1996). Requirement for Ceramide-Initiated SAPK/JNK Signalling in Stress-Induced Apoptosis. Nature.

[9] Kohen R. (1999). Skin Antioxidants: Their Role in Aging and in Oxidative Stress--New Approaches for Their Evaluation. Biomed. Pharmacother. Biomedecine Pharmacother..

[10] Dadej I., Wołowiec J. (2003). The Role of UVA of the Skin Pathology. Adv. Dermatol. Allergol. Dermatol. Alergol..

[11] Battie C., Jitsukawa S., Bernerd F., Del Bino S., Marionnet C., Verschoore M. (2014). New Insights in Photoaging, UVA Induced Damage and Skin Types. Exp. Dermatol..

[12] Vostalova J., Rajnochova Svobodova A., Galandakova A., Sianska J., Dolezal D., Ulrichova J. (2013). Differential Modulation of Inflammatory Markers in Plasma and Skin after Single Exposures to UVA or UVB Radiation in Vivo. Biomed. Pap. Med. Fac. Univ. Palacky Olomouc Czechoslov..

[13] Huang Z.-R., Lin Y.-K., Fang J.-Y. (2009). Biological and Pharmacological Activities of Squalene and Related Compounds: Potential Uses in Cosmetic Dermatology. Molecules.

[14] Oliveira A.L.S., Valente D., Moreira H.R., Pintado M., Costa P. (2022). Effect of Squalane-Based Emulsion on Polyphenols Skin Penetration: Ex Vivo Skin Study. Colloids Surf. B Biointerfaces.

[15] Barp L., Miklavčič Višnjevec A., Moret S. (2024). Analytical Determination of Squalene in Extra Virgin Olive Oil and Olive Processing By-Products, and Its Valorization as an Ingredient in Functional Food—A Critical Review. Molecules.

[16] (1990). Final Report on the Safety Assessment of Squalane and Squalene. J. Am. Coll. Toxicol..

[17] Wolosik K., Chalecka M., Palka J., Surazynski A. (2023). Protective Effect of Amaranthus Cruentus L. Seed Oil on UVA-Radiation-Induced Apoptosis in Human Skin Fibroblasts. Int. J. Mol. Sci..

[18] Ibrahim N.I., Naina Mohamed I. (2021). Interdependence of Anti-Inflammatory and Antioxidant Properties of Squalene–Implication for Cardiovascular Health. Life.

[19] Jariashvili K., Madhan B., Brodsky B., Kuchava A., Namicheishvili L., Metreveli N. (2012). Uv Damage of Collagen: Insights from Model Collagen Peptides. Biopolymers.

[20] Wolosik K., Chalecka M., Palka J., Mitera B., Surazynski A. (2024). Amaranthus Cruentus L. Seed Oil Counteracts UVA-Radiation-Induced Inhibition of Collagen Biosynthesis and Wound Healing in Human Skin Fibroblasts. Int. J. Mol. Sci..

[21] Afaq F., Malik A., Syed D., Maes D., Matsui M.S., Mukhtar H. (2005). Pomegranate Fruit Extract Modulates UV-B-Mediated Phosphorylation of Mitogen-Activated Protein Kinases and Activation of Nuclear Factor Kappa B in Normal Human Epidermal Keratinocytes Paragraph Sign. Photochem. Photobiol..

[22] Katiyar S.K., Matsui M.S., Elmets C.A., Mukhtar H. (1999). Polyphenolic Antioxidant (-)-Epigallocatechin-3-Gallate from Green Tea Reduces UVB-Induced Inflammatory Responses and Infiltration of Leukocytes in Human Skin. Photochem. Photobiol..

[23] Rippe R.A., Schrum L.W., Stefanovic B., Solis-Herruzo J.A., Brenner D.A. (1999). NF-kappaB Inhibits Expression of the Alpha1(I) Collagen Gene. DNA Cell Biol..

[24] O’Dea E.L., Kearns J.D., Hoffmann A. (2008). UV as an Amplifier Rather than Inducer of NF-κB Activity. Mol. Cell.

[25] Ryšavá A., Vostálová J., Rajnochová Svobodová A. (2021). Effect of Ultraviolet Radiation on the Nrf2 Signaling Pathway in Skin Cells. Int. J. Radiat. Biol..

[26] Gęgotek A., Jastrząb A., Jarocka-Karpowicz I., Muszyńska M., Skrzydlewska E. (2018). The Effect of Sea Buckthorn (Hippophae Rhamnoides L.) Seed Oil on UV-Induced Changes in Lipid Metabolism of Human Skin Cells. Antioxidants.

[27] Darr D., Fridovich I. (1994). Free Radicals in Cutaneous Biology. J. Invest. Dermatol..

[28] Poon F., Kang S., Chien A.L. (2015). Mechanisms and Treatments of Photoaging. Photodermatol. Photoimmunol. Photomed..

[29] Carmichael J., DeGraff W.G., Gazdar A.F., Minna J.D., Mitchell J.B. (1987). Evaluation of a Tetrazolium-Based Semiautomated Colorimetric Assay: Assessment of Chemosensitivity Testing. Cancer Res..

[30] Peterkofsky B., Diegelmann R. (1971). Use of a Mixture of Proteinase-Free Collagenases for the Specific Assay of Radioactive Collagen in the Presence of Other Proteins. Biochemistry.

[31] Myara I., Charpentier C., Lemonnier A. (1982). Optimal Conditions for Prolidase Assay by Proline Colorimetric Determination: Application to Iminodipeptiduria. Clin. Chim. Acta.

[32] Lowry O.H., Rosebrough N.J., Farr A.L., Randall R.J. (1951). Protein Measurement with the Folin Phenol Reagent. J. Biol. Chem..

[33] Laemmli U.K. (1970). Cleavage of Structural Proteins during the Assembly of the Head of Bacteriophage T4. Nature.

