# Supplementary material for: Squalane as a Promising Agent Protecting UV-Induced Inhibition of Collagen Biosynthesis and Wound Healing in Human Dermal Fibroblast
Source: Molecules. 2025 Apr 29;30(9):1964. doi: 10.3390/molecules30091964 (PMC12073650; doi:10.3390/molecules30091964)

**Table S1** The MTT test for HDF viability **(A)** treated with Sq at concentrations of 0.005%, 0.01% and 0.015%, 0.02% and 0.025% vs. the control **(B)** irradiated with UVA and treated with Sq at concentrations of 0.005%, 0.01% and 0.015% vs. the control. The mean values. standard deviation (SD), standard error (SEM) and median (Me) from the 3 experiments. *Statistically significant differences at $p < 0.05$ compared with the control. ## Statistically significant differences at $p < 0.05$ compared with the UVA group.

**(A)**

| | I | II | III | Mean Value | % Value | SD | SEM | Me |
|---|---|---|---|---|---|---|---|---|
| **Control** | 0.794 | 0.789 | 0.74 | 0.774 | 100 | 0.0298 | 0.023 | 0.789 |
| **Sq 0.005%** | 0.683 | 0.764 | 0.7 | 0.716 | 92.463 | 0.0427 | 0.0322 | 0.7 |
| **Sq 0.01%** | 0.77 | 0.778 | 0.798 | 0.782 | 101.033 | 0.0144 | 0.0106 | 0.778 |
| **Sq 0.015%** | 0.791 | 0.876 | 0.748 | 0.805 | 104.00 | 0.0651 | 0.0473 | 0.791 |
| **Sq 0.02%** | 0.615 | 0.561 | 0.64 | 0.605 | 78.208* | 0.0403 | 0.0295 | 0.615 |
| **Sq 0.025%** | 0.51 | 0.672 | 0.554 | 0.578 | 74.763* | 0.0837 | 0.062 | 0.554 |

**(B)**

| | I | II | III | Mean Value | % Value | SD | SEM | Me |
|---|---|---|---|---|---|---|---|---|
| **Non-UVA-Treated Cells (– UVA)** | | | | | | | | |
| Control | 0.794 | 0.789 | 0.74 | 0.774 | 100 | 0.029838 | 0.023 | 0.789 |
| **Sq 0.005%** | 0.683 | 0.764 | 0.7 | 0.716 | 92.463 | 0.04271 | 0.0322 | 0.7 |
| **Sq 0.01%** | 0.77 | 0.778 | 0.798 | 0.782 | 101.033 | 0.01442 | 0.0106 | 0.778 |
| **Sq 0.015%** | 0.791 | 0.876 | 0.748 | 0.805 | 104.00 | 0.06513 | 0.0473 | 0.791 |
| **UVA-Treated Cells (+ UVA)** | | | | | | | | |
| **UVA** | 0.449 | 0.413 | 0.425 | 0.429 | 55.426* | 0.01833 | 0.0133 | 0.425 |
| **Sq 0.005%** | 0.473 | 0.544 | 0.473 | 0.496 | 64.1688 | 0.04099 | 0.0315 | 0.473 |
| **Sq 0.01%** | 0.51 | 0.672 | 0.554 | 0.579 | 74.763 | 0.08376 | 0.0622 | 0.554 |
| **Sq 0.015%** | 0.615 | 0.561 | 0.64 | 0.605 | 78.208## | 0.04037 | 0.0295 | 0.615 |

**Table S2** Collagen biosynthesis measurements in HDF irradiated with UVA and treated with Sq at concentrations of 0.005%, 0.01% and 0.015%. (1$^{st}$ - first measurement in sample. 2$^{nd}$ - second measurement in sample, Δ - difference between 1st and 2nd measurement in sample, *statistically significant differences at $p < 0.05$ compared to the control.

| **L-5-[3H]-proline incorporation into collagen proteins** | | | | | | | | | | | | | |
|---|---|---|---|---|---|---|---|---|---|---|---|---|---|
| **Sample** | **1st** | **2nd** | **$\Delta^1$** | **1st** | **2nd** | **$\Delta^2$** | **1st** | **2nd** | **$\Delta^3$** | **Mean value ($\Delta^1$+ $\Delta^2$+ $\Delta^3$)/3** | **% per cent** | **SD** | **SEM** |
| **Non-UVA-Treated Cells (– UVA)** | | | | | | | | | | | | | |
| **Control** | 2486 | 1503 | **983** | 1849 | 1682 | **167** | 1866 | 1863 | **3** | 384.33 | 100 | 832.47 | 277.49 |
| **Sq 0.005%** | 1881 | 1358 | **523** | 2116 | 1761 | **355** | 2116 | 1861 | **255** | 377.66 | 98.26 | 771.19 | 257.064 |
| **Sq 0.01%** | 1539 | 1149 | **390** | 1571 | 1334 | **237** | 1536 | 1090 | **446** | 357.66 | 93.06 | 535.89 | 178.63 |
| **Sq 0.015%** | 1782 | 1084 | **698** | 2228 | 1823 | **405** | 1800 | 1780 | **20** | 374.33 | 99.11 | 766.09 | 255.36 |
| **UVA-Treated Cells (+ UVA)** | | | | | | | | | | | | | |
| **UVA** | 914 | 616 | **298** | 846 | 681 | **165** | 685 | 517 | **168** | 210.33 | 54.72* | 277.98 | 92.66 |
| **Sq 0.005%** | 1506 | 1260 | **246** | 1558 | 1350 | **208** | 1454 | 1121 | **333** | 262.33 | 68.25 | 572.14 | 190.71 |
| **Sq 0.01%** | 1505 | 1018 | **487** | 1189 | 1121 | **68** | 1594 | 1350 | **244** | 266.33 | 69.29 | 555.02 | 185.00 |
| **Sq 0.015%** | 1182 | 948 | **234** | 1156 | 1035 | **121** | 1400 | 862 | **538** | 297.66 | 77.45 | 440.90 | 146.96 |

**Table S3** Prolidase activity in HDF irradiated with UVA and treated with Sq at concentrations of 0.005%, 0.01% and 0.015%. The mean values from the experiments performed in triplicates, *statistically significant differences at $p < 0.05$ compared with the control, ##statistically significant difference at $p < 0.05$ compared to UVA.

| **The nanomoles of proline released from the synthetic substrate (Gly-Pro)** | | | | | | | | |
|---|---|---|---|---|---|---|---|---|
| **Sample** | **Control** | **Sq 0.005%** | **Sq 0.01%** | **Sq 0.015%** | **UVA** | **Sq 0.005% + UVA** | **Sq 0.01% + UVA** | **Sq 0.015% + UVA** |
| **Mean value** | 116.4 | 110.58 | 114.072 | 124.54 | 62.85 | 109.42 | 112.9 | 113.72 |
| **% of control value** | 100 | 95 | 98 | 107 | 54* | 94## | 97## | 98## |

**Figure S1** Western blot densitometry (plot measurement) results by gel analysis tool for Fiji ImageJ® 2.16.0 for **(A)** β1- integrin **(B)** and IGF-IR receptors expression in UVA-irradiated HDF in the presence of Sq at concentrations of 0.005%, 0.01%, and 0.015%.

**(A)**

| Sample | Plot measurement | % of control |
|---|---|---|
| **Control (a)** | 75942.378 | 100 |
| **Sq 0.005% (b)** | 62877.931 | 82.79689504 |
| **Sq 0.01% (c)** | 71827.411 | 94.58146149 |
| **Sq 0.015% (d)** | 71818.976 | 94.57035438 |
| **UVA (e)** | 42621.751 | 56.1238035 |
| **Sq 0.005% + UVA (f)** | 64818.562 | 85.35229434 |
| **Sq 0.01% + UVA (g)** | 54809.813 | 72.17289535 |
| **Sq 0.015% + UVA (h)** | 68639.66 | 90.38386973 |

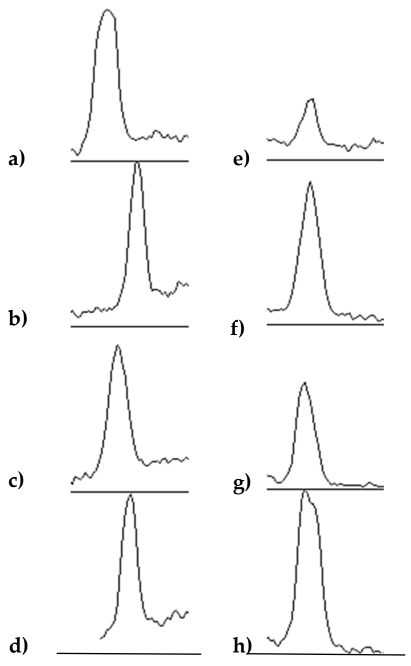


**(B)**

| Sample | Plot measurement | % of control |
|---|---|---|
| **Control (a)** | 12505.217 | 100 |
| **Sq 0.005% (b)** | 11557.731 | 92 |
| **Sq 0.01% (c)** | 11592.489 | 93 |
| **Sq 0.015% (d)** | 11684.903 | 93 |
| **UVA (e)** | 5374.740 | 43 |
| **Sq 0.005% + UVA (f)** | 6436.933 | 51 |
| **Sq 0.01% + UVA (g)** | 6621.861 | 53 |
| **Sq 0.015% + UVA (h)** | 9098.690 | 73 |

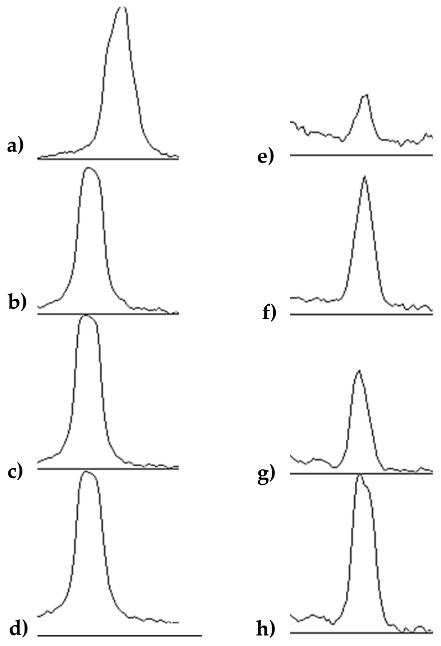

**Figure S2** Western blot densitometry (plot measurement) results by gel analysis tool for ImageJ® 1.8.0 for **(A)** TGF-β1 and **(B)** p38 protein expression in UVA-irradiated HDF in the presence of Sq at concentrations of 0.005%, 0.01%, and 0.015%.

**(A)**

| Sample | Plot measurement | % of control |
|---|---|---|
| **Control** (a) | 7292.711 | 100 |
| **Sq 0.005%** (b) | 6128.004 | 84 |
| **Sq 0.01%** (c) | 7395.640 | 101 |
| **Sq 0.015%** (d) | 7937.861 | 108 |
| **UVA** (e) | 2320.347 | 32 |
| **Sq 0.005% + UVA** (f) | 5222.146 | 72 |
| **Sq 0.01% + UVA** (g) | 5162.033 | 71 |
| **Sq 0.015% + UVA** (h) | 6499.740 | 88 |

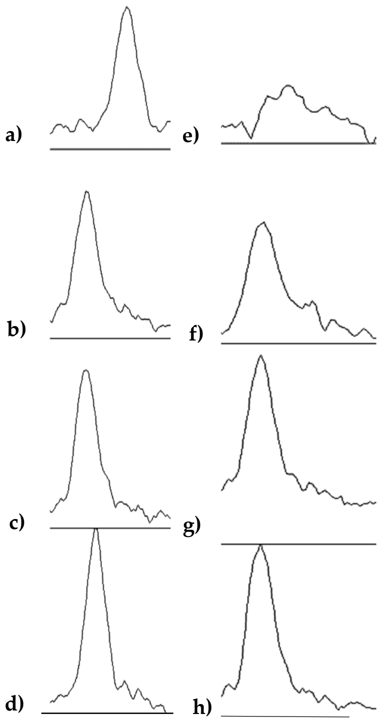


**(B)**

| Sample | Plot measurement | % of control |
|---|---|---|
| **Control** (a) | 8835.489 | 100 |
| **Sq 0.005%** (b) | 4994.468 | 56 |
| **Sq 0.01%** (c) | 7657.296 | 87 |
| **Sq 0.015%** (d) | 8421.317 | 95 |
| **UVA** (e) | 10855.560 | 123 |
| **Sq 0.005% + UVA** (f) | 5716.560 | 65 |
| **Sq 0.01% + UVA** (g) | 6782.560 | 77 |
| **Sq 0.015% + UVA** (h) | 6143.167 | 70 |

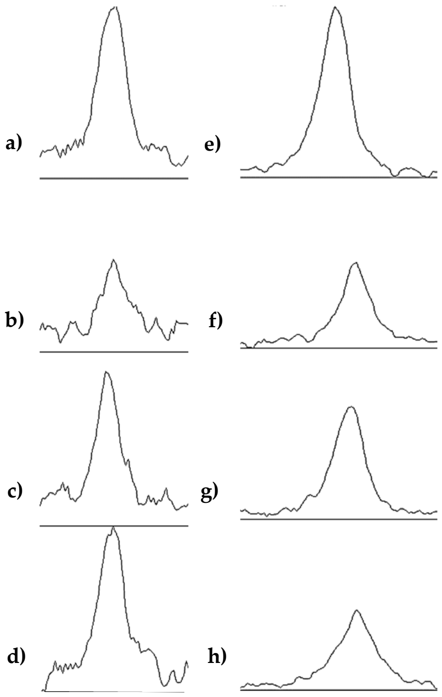

**Figure S3** Western blot densitometry (plot measurement) results by gel analysis tool for ImageJ® 1.8.0 for COX-2 protein expression in UVA-irradiated HDF in the presence of Sq at concentrations of 0.005%, 0.01%, and 0.015%.

| Sample | Plot measurement | % of control |
|---|---|---|
| **Control (a)** | 6096.225 | 100 |
| **Sq 0.005% (b)** | 3308.740 | 54 |
| **Sq 0.01% (c)** | 4736.004 | 78 |
| **Sq 0.015% (d)** | 3756.983 | 62 |
| **UVA (e)** | 9247.652 | 152 |
| **Sq 0.005% + UVA (f)** | 5579.095 | 92 |
| **Sq 0.01% + UVA (g)** | 5866.368 | 96 |
| **Sq 0.015% + UVA (h)** | 6005.196 | 99 |

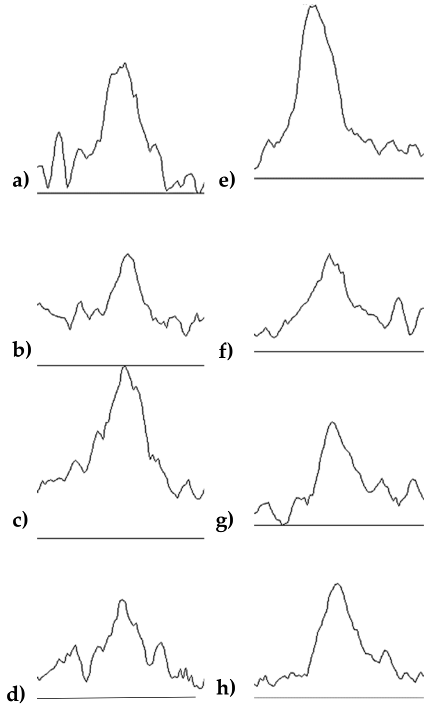

Supplement: Supplementary file 1 [file molecules-30-01964-s001.zip › molecules-3566168-supplementary.pdf]
